# Health Anxiety and Its Relationship to Thyroid-Hormone-Suppression Therapy in Patients with Differentiated Thyroid Cancer

**DOI:** 10.3390/cancers14102349

**Published:** 2022-05-10

**Authors:** Maximilian Zoltek, Therese M.-L. Andersson, Erland Axelsson, Christel Hedman, Catharina Ihre Lundgren

**Affiliations:** 1 Department of Molecular Medicine and Surgery, Karolinska Institutet, 17176 Stockholm, Sweden; christel.hedman@ki.se (C.H.); cia.ihre-lundgren@ki.se (C.I.L.); 2 Department of Medical Epidemiology and Biostatistics, Karolinska Institutet, 17176 Stockholm, Sweden; therese.m-l.andersson@ki.se; 3 Department of Neurobiology, Care Sciences and Society, Karolinska Institutet, 14152 Huddinge, Sweden; erland.axelsson@ki.se; 4 Department of Clinical Neuroscience, Karolinska Institutet, 17165 Solna, Sweden; 5 Liljeholmen Primary Health Care Centre, Region Stockholm, 11763 Stockholm, Sweden; 6 Academic Primary Health Care Centre, Region Stockholm, 11365 Stockholm, Sweden; 7 Research and Development Department, Stockholms Sjukhem Foundation, Mariebergsgatan 22, 11219 Stockholm, Sweden; 8 Division of Palliative Care, Department of Clinical Sciences Lund, Lund University, Scheelevägen 2, Medicon Village, 22381 Lund, Sweden; 9 Department of Breast, Endocrine Tumours and Sarcoma, Karolinska University Hospital, 17176 Stockholm, Sweden

**Keywords:** health anxiety, differentiated thyroid cancer, thyroid-stimulating hormone

## Abstract

**Simple Summary:**

Differentiated thyroid cancer (DTC) has a good prognosis; however, patients often need lifelong follow up and face potentially chronic psychiatric problems, such as health anxiety. We investigated the relationship between health anxiety and TSH-suppression treatment, which many patients receive lifelong. The health anxiety was assessed by using the 14-item Short Health Anxiety Inventory (SHAI-14), which is scored 0–42; higher values indicate more health anxiety. Out of 146 patients with DTC, 24 (16%) had clinically significant health anxiety. Patients with TSH levels of 0.1–0.5 (mE/L) scored, on average, 3.28 points more (*p*-value 0.01) on the SHAI-14 compared to patients with TSH levels > 0.5. We found that health anxiety appears to be slightly more common among DTC patients compared to the general population, but this was not clearly connected to the TSH-suppression treatment.

**Abstract:**

Differentiated thyroid cancer (DTC) has a good prognosis; however, patients often need lifelong follow up, and they face potential side effects. The aim of this study was to investigate health anxiety among DTC patients and its relationship to TSH suppression. In 2020, patients from a previous cohort who were from Stockholm completed the 14-item Short Health Anxiety Inventory (SHAI-14; 0–42; 18 being the threshold for clinical significance) and a study-specific questionnaire. Clinical information was also retrieved from medical records. Linear regression was used to investigate the relationship between the TSH levels and the SHAI-14, while adjusting for potential confounders. In total, 146 (73%) patients were included. A total of 24 respondents (16%) scored 18 or more on the SHAI-14, and the mean score was 11.3. Patients with TSH levels of 0.1–0.5 (mE/L) scored, on average, 3.28 points more (*p*-value 0.01) on the SHAI-14 compared to patients with TSH levels > 0.5. There was no statistically significant difference between patients with TSH levels < 0.1 and TSH levels > 0.5. Thus, we found no linear relationship between the TSH values and health anxiety. Clinically significant levels of health anxiety are slightly higher than those in the general population, but do not appear to be a major psychiatric comorbidity among patients with DTC.

## 1. Introduction

Differentiated thyroid cancer (DTC) accounts for more than 95% of all thyroid carcinomas [1], and patients face a good prognosis, with a 10-year overall survival that exceeds 90% [2]. Curative treatment consists of thyroid surgery, a total thyroidectomy, or lobectomy, in some cases, and radioactive iodine treatment when needed, on the basis of the risk of recurrence [3,4]. After a thyroidectomy, patients are dependent on lifelong thyroid hormone supplementation. Because thyroid-stimulating hormone (TSH) stimulates cancer cells [1,4], patients usually receive levothyroxine in doses to suppress the TSH. This induces a state of iatrogenic subclinical hyperthyroidism, and a more aggressive suppression therapy reduces the risk of recurrence, at least in patients in whom this risk is the highest [3,5]. In Sweden, patients with thyroid cancer are given treatment according to the national guidelines [6], which are based on recommendations from the European Thyroid Association (ETA) and the American Thyroid Association (ATA) [3,7]. The TSH-suppressing treatment is known to produce side effects, such as osteoporosis and atrial fibrillation [8,9,10].

Although most patients adapt to life after a cancer diagnosis and its treatment, some develop chronic mental health problems [11,12], which could partly depend on the fear of cancer recurrence. With regard to DTC, despite the overall favorable prognosis, recurrence many years after treatment is not uncommon [13]. Thus, the fear of cancer recurrence and the reduced quality of life seen in DTC patients may persist, and it is predictive of healthcare utilization [14] for more than a decade postdiagnosis [15]. A recent systematic review reports a correlation between anxiety levels and thyroid hormone disturbance in benign thyroid disease. Some studies highlight a negative correlation between TSH levels and anxiety disorders; however, they did not include cancer patients [16]. Patients with DTC are thus prone to anxiety disorders by two potential pathways: psychological, and physiological (due to TSH suppression).

Health anxiety is a widely researched and relatively stable pattern of cognition, emotion, and behavior that is characterized by the fear or preoccupation with having or developing a serious disease [17,18]. Individuals with clinically significant health anxiety are quick to respond to health cues, and they commonly interpret minor bodily sensations (e.g., palpitations, increased perspiration, dyspnea) and trivial physical abnormalities (e.g., skin discoloration, light-headedness) as signs of a serious somatic disease [19]. This often results in a behavioral pattern where patients repeatedly seek reassurance from healthcare professionals, usually with little or no relief, except in the very short term. It is widely believed that the experience of having been diagnosed with a potentially serious disease, such as DTC, or of having been exposed to demanding medical treatment, such as surgical procedures, increases the risk of developing very high and persistent levels of health anxiety over the lifespan [20]. The experimental research also indicates that frequent engagement in health behaviors such as body checking and reassurance seeking—which are behaviors that are commonly promoted in routine cancer care—may increase health-anxiety levels, at least in the short term [21]. Clinically significant health anxiety is associated with substantial suffering for the individual [22], and it poses a considerable economic burden that is comparable to those of many chronic medical conditions [23]. The comorbidity rates with other psychiatric problems are considerable, as is illustrated by a 2007 survey of the general population of Australia that reports odds ratios of 5.77 (95% CI: 4.61–7.23) for anxiety disorders, and 4.66 (3.42–6.34) for major depressive disorder [24]. However, the majority of patients with health anxiety respond to cognitive-behavior therapy, which also has an effect on the secondary symptoms of general anxiety and depression [25]. Promising effects have also been reported in medical patients, with sustained effects up to 8 years [26]. We note that, tentatively, the existing treatment protocols for the fear of cancer recurrence appear to have more limited effects [27] than typical treatments with the aim of reducing the pathological levels of health anxiety as they are traditionally conceptualized in the psychiatric context [25,27]. In order to further inform tailored clinical interventions and the development of more efficacious psychological treatments, it is important to understand whether health anxiety is a core characteristic of long-term psychiatric problems that are seen in DTC.

To our knowledge, no previous studies have investigated health anxiety among patients with DTC, or its potential connection to TSH suppression. The aims of this study were to investigate whether there is a negative relationship between TSH levels and health anxiety, and to investigate the prevalence of health anxiety in DTC patients.

## 2. Materials and Methods

### 2.1. Design and Recruitment

This cross-sectional cohort study was based on a population-based nationwide cohort study on the health-related quality of life in patients with DTC in Sweden [28]. In the nationwide study, from all Swedish oncology departments, patients diagnosed between 2012 and 2017 that had received surgery and radioactive iodine treatment for DTC were asked to participate (349 out of 487 eligible patients were included; response rate: 72%). Inclusion criteria were: an age ≥18 years at diagnosis, Swedish language proficiency, and a primary diagnosis of DTC. Exclusion criteria were: small DTC (T1a), unplanned radioactive iodine treatment; ongoing treatment for other malignancies; anaplastic or medullary thyroid cancer; or the recurrence of DTC at inclusion. Patients were invited by mail, and by completing and returning the questionnaires, they consented to their participation, and to the use of their data for research purposes.

From this cohort, in the present study, we invited all patients from the Stockholm area that were followed up at Karolinska University Hospital (*n* = 201) during the year of 2020 to participate, regardless of duration from diagnosis. Patients received a letter describing the study, together with a survey package for the present study (please see below), and reminders were sent no more than two times. Written informed consent was obtained from all participants. Ethical approval for the study was obtained from the Regional Ethical Review Board of Stockholm and from the Swedish Ethical Review Authority (2011/718-31/2; 2011/1847-32; 2020-02722).

### 2.2. Self-Report Questionnaires

The survey package included the 14-item Short Health Anxiety Inventory (SHAI-14), as a measure of the health anxiety [18], and a study-specific questionnaire. The SHAI-14 is widely considered to exhibit adequate psychometric properties, including internal consistency, test–retest reliability, and convergent and discriminant validity [29]. For each item on the SHAI-14, the respondent chooses among four sentences that range from, for example, “I do not worry about my health” to “I spend most of my time worrying about my health”. Each item is scored on a 4-point Likert scale from 0 to 3, which results in a sum score range of 0–42; higher scores, being indicative of more health anxiety, and a score of 18 or higher, is widely considered to be indicative of clinically significant health anxiety [30]. Whenever multiple answers were given to the same SHAI-14 item, the highest alternative was used. Patients were dropped from the analysis if they omitted three or more items. For patients with 1 or 2 missing items, the mean value from the other items was imputed. The study-specific questionnaire included questions regarding information on socioeconomic background, including the educational level, occupation, and civil status, and disease-specific questions regarding symptoms and relapse in thyroid cancer. Comorbidities diagnosed by a physician were self-reported, and they were subsequently grouped into “none to one”, or “at least two”.

### 2.3. Histological and Laboratory Parameters

The tumor stage (TNM), histopathology report, and information on the primary treatment modality for DTC were collected at diagnosis (surgery, radioactive iodine, external radiation). Information about biomarkers were collected from medical records (TSH, Thyroglobulin (Tg), Tg-antibodies) at the time of inclusion in this study. In addition, information on recurrent or persistent disease was collected from medical records at the time of inclusion.

TSH values (all TSH units stated in mE/L) were categorized into three levels: “unsuppressed” (TSH > 0.5); “mildly suppressed” (TSH: 0.1–0.5); and “suppressed” (TSH < 0.1), according to the Swedish national guidelines [6]. We defined persistence or recurrence in cancer disease as a Tg value exceeding 2, or additional intervention with radioactive iodine, external radiation, or surgical intervention post primary treatment. In this study, we defined any TNM stage containing T4, T3, N1, or M1 as “high risk” thyroid cancer; all others were classified as “low risk” thyroid cancer, as per the Swedish national guidelines [6].

### 2.4. Statistical Analysis

Patient characteristics were described by using standard descriptive statistics (Table 1). To investigate the effect of the level of TSH on health anxiety, we employed a multiple linear regression model. Health anxiety (the SHAI-14; 0–42) was regressed on the TSH groups (TSH < 0.1, TSH 0.1–0.5, and TSH > 0.5), the recurrence (yes/no), the risk status (low/high), and the comorbidity (one or less/two or more). We calculated Cohen’s d for the effect of the TSH level on the basis of the coefficients from the linear regression in order to obtain a standardized mean difference, which is easier to interpret in terms of practical relevance. Absolute values for d around 0.2 are usually regarded as small, 0.5 as moderate, and 0.8 as large [31]. Stata 17, StataCorp LP Lakeway Drive, Texas, USA, was used for the statistical analyses.

## 3. Results

### 3.1. Sociodemographic and Clinical Characteristics

Out of 201 eligible study participants, 146 (73%) were included in the final cohort (152 answered the study questionnaires, and 6 persons were excluded due to three or more missing answers on the SHAI-14). Most were female (73%), and the age distribution was tilted toward the young, where 45% were less than 45 years old at diagnosis, and 35% had been diagnosed between 45–65 years of age (Table 1). A total of 60% had completed university education, and 29% reported that they had completed high school education. Most of the responders were either working (61%) or retired (31%). With regard to the TNM classification, the distribution among T stage I-III was about 30% for the respective stage, and only 4% were staged T-stage IV. Approximately half of the patients (53%) had lymph node metastases, and only two patients had been diagnosed with distant metastases. Most patients (68%) had their cancer disease classified as “high risk” at the time of diagnosis, and 15% had experienced a recurrence in their cancer disease. The majority of patients had less than two comorbidities when answering the questionnaires (73%). The median time that passed from diagnosis to answering questionnaires was 6 years.

### 3.2. Distribution of Health Anxiety in DTC Patients

In total, 24 respondents (16%) scored 18 points or more on the SHAI-14, which corresponds to clinically significant health anxiety. The mean score on the SHAI-14 among respondents was 11.3, and women scored, on average, slightly higher than men (12.5 compared to 8.3). With regard to the age distribution, the average SHAI-14 scores were similar in all age categories (Table 2).

### 3.3. Effect of Thyroid-Stimulating Hormone on the Level of Health Anxiety

In the primary analysis, the health anxiety (the SHAI-14) was regressed on the TSH category (Table 3), and was adjusted for the risk group, the comorbidities, and the recurrence in thyroid cancer. The results indicate that patients with TSH levels of 0.1–0.5 scored, on average, 3.28 points higher on the SHAI-14 compared to those with TSH levels above 0.5 (*p* < 0.01). Cohen’s d for this difference was 0.54. Patients with TSH levels < 0.1 scored, on average, 0.96 points higher on the SHAI-14 compared to the unsuppressed group; however, this value was not statistically significant (*p* = 0.48). Cohen’s d for this difference was 0.15.

## 4. Discussion

This cohort study is, to the best of our knowledge, the first to investigate the effect of TSH levels on health anxiety, and the prevalence of health anxiety among patients with DTC. It has previously been found that the consequences of health anxiety can be substantial for patients, as well as for society. Patients with clinically significant health anxiety have higher levels of health impairment [32], increased risk of long-term sick leave [33], and the condition is significantly associated with increased consumption of both somatic and mental healthcare services [34]. A small increase in health anxiety has also been found to raise the risk of ischemic heart disease [35], and it is therefore essential to identify patients who suffer from clinically significant health anxiety.

We investigated the prevalence of clinically significant health anxiety, and found it to be approximately 16%, on the basis of the cutoff of 18 points on the SHAI-14. This figure can be compared to other cutoff-based prevalence figures, such as those presented by Tyrer et al. [36], who, by using a slightly higher cutoff of 20 on the SHAI-14, found that about 1 in 5 patients in British medical clinics scored above the threshold. On the basis of the same cutoff, another study found a prevalence of 23% in self-recruited women with breast cancer [37]. The mean SHAI-14 score in our study was 11.3, which appears to be slightly higher than for healthy controls [18], but is clearly lower than the mean of 15.5 that is seen in self-recruited women with breast cancer [37]. The relatively substantial number of patients who scored above the cutoff, despite a relatively low mean level of health anxiety, is mirrored by the fact that the standard deviation of 6.5 appears to have been unusually high, as compared to most other samples [18]. By considering that the mean time from cancer diagnosis, in this sample, was 6 years, it appears that, for a noteworthy proportion of patients with DTC who experience significant levels of health anxiety, this problem might not resolve, even in the long term, without specific clinical intervention.

Our main finding is that TSH suppression does not appear to be clearly predictive of health anxiety, at least not in a linear fashion. The patient group with the most suppressed TSH levels (<0.1) did not differ significantly from the group with no TSH suppression, and the effect size was small (d = 0.15). However, the group with mild TSH suppression (0.1–0.5) had a significantly higher level of health anxiety than the group with unsuppressed TSH levels, after adjusting for the risk group, the comorbidities, and the recurrence in cancer disease. The effect size was moderate (d = 0.54). This pattern was unexpected but not unprecedented. TSH suppression has previously been thought to be part of the symptoms that are related to lower quality of life in patients with DTC [38,39]; however, more recent studies have found no linear relationship [28,40].

Higher levels of health anxiety in mildly suppressed patients are possibly best understood from a psychological perspective, which is difficult to capture in a linear regression analysis, at least by using the variables that are available here; qualitative research could shed more light on the topic. A portion of those with mild suppression could be patients that are followed for long intervals due to the low risk of recurrence, and the missing security from regular checkups might increase health anxiety. Furthermore, some of the individuals among the mildly suppressed patients might have been the ones with pronounced symptoms from complete TSH suppression, and they may therefore have been prescribed a lower dose of levothyroxine, which could partly be a possible explanation for the higher levels of health anxiety in this group. Our results are in line with previous literature, and they raise further questions about the role of TSH suppression in relation to psychopathology, which suggests that it is probably not the most important variable that underlies mental health problems in patients with DTC.

In summary, patients with DTC appear to have slightly heightened average levels of health anxiety, although this was not substantially burdensome on a population basis. The increased levels of health anxiety could partly be related to the fear of recurrence, which is known to be as high, or even higher, in patients with DTC compared to patients with a cancer diagnosis with worse prognosis. As cancer patients with fear of recurrence are more prone to consult healthcare providers, and also have increased use of psychotropic medications [41], these behaviors overlap with those of health anxiety.

The strengths of this study include the open access to medical records and laboratory data. The health anxiety was assessed by using the SHAI-14, which is a widespread and psychometrically sound instrument [18]. In the current literature, the variants of the SHAI are probably the closest to the gold-standard measure of health anxiety that one can come, and these have been used as the primary outcome estimates in several clinical trials [26,42,43,44]. Furthermore, information on the recurrence in cancer disease, the TSH values, and the TNM classification could be retrieved from medical records. For comorbidities, a limitation is that we had to rely on self-reported diagnoses. A general limitation of a survey study is also selection bias. In this case, conceivably, the patients’ levels of health anxiety could have been predictive of whether or not they completed the questionnaires that were required for this study. Other limitations of the study include the variability in the TSH measurement in time. The TSH values were retrieved from medical records, and the closest value prior to answering the questionnaires was selected, where the median was 180 days. The median duration from diagnosis was 6 years and, assuming stability in cancer disease, one is to expect that most patients did not have their TSH targets adjusted less than half a year before answering the questionnaires.

## 5. Conclusions

This is, to the best of our knowledge, the first study to report on health anxiety in patients with DTC. Clinically significant levels of health anxiety are probably slightly higher than the ones seen in the general population. The levels of TSH suppression were not predictive of health anxiety in a linear fashion among DTC patients with long-term follow up. A sub portion of DTC patients may benefit from treatment for health anxiety; however, the symptom levels were not high enough to be considered a major psychiatric comorbidity in general in the long-term follow up.

## Figures and Tables

**Table 1:** Clinical characteristics of study population with differentiated thyroid cancer.

	N	(%)
Total	146	(100)
Women	107	(73)
Men	39	(27)
Age		
<45 years	65	(45)
45–65 years	51	(35)
65+ years	30	(21)
Tumor (T) Stage		
0	1	(1)
I	50	(34)
II	43	(29)
III	48	(33)
IV	4	(3)
Lymph-Node-Metastases (N) Stage		
0	20	(14)
I	77	(53)
X	49	(34)
Distant-Metastases (M) Stage		
0	11	(8)
I	2	(1)
X	133	(91)
TSH suppression		
TSH < 0.1	39	(27)
TSH 0.1–0.5	47	(32)
TSH > 0.5	60	(41)
Risk Staging		
High-risk cancer	100	(68)
Low-risk cancer	46	(32)
Number of Concomitant Diseases		
Two or more	40	(27)
Less than two	106	(73)
Thyroid Cancer Recurrence		
Yes	22	(15)
No	124	(85)
Time Since Diagnosis, in Years		
Mean	6.31	
Median	6.05	
Min	3.61	
Max	9.11	

TSH: thyroid-stimulating hormone. TNM (tumor stage, lymph node metastases, distant metastases) classification and risk staging were collected at time of cancer diagnosis. Recurrence in thyroid cancer was considered from diagnosis to inclusion. Remaining variables were retrieved at time of inclusion. TNM stage containing T4, T3, N1, and M1 was defined as “high risk” thyroid cancer; all others were classified as “low risk” thyroid cancer.

**Table 2:** Distribution of 14-item Short-Health-Anxiety-Inventory scores.

	Mean (SD), Range
Total Sample	11.3 (6.5), 0–33
Gender	
Female	12.5 (6.4), 0–29
Male	8.3 (6.0), 0–33
Age	
<45 years	11.6 (5.8), 0–26
45–65 years	11.4 (6.2), 1–29
65+ years	10.7 (8.4), 0–33
Clinically Significant (≥18 points)	*n* = 24 (16%)

SD: standard deviation. The 14-item Short Health Anxiety Inventory (SHAI-14) has a theoretical range of 0–42. The cutoff for clinically significant health anxiety is 18 points.

**Table 3:** Levels of TSH and other clinical variables, and the severity of anxiety in patients with DTC. Multivariate analysis.

	Coefficient	95% CI	*p*-Value
Intercept	10.83	8.54–13.13	0.00
TSH < 0.1	0.96	−1.72–3.64	0.48
TSH 0.1–0.5	3.28	0.82–5.75	0.01
TSH >0.5	Ref	-	-
Low risk	Ref	-	-
High risk	−2.03	−4.39–0.32	0.09
Comorbidities: 0–1	Ref	-	-
Comorbidities: 2 or more	1.84	−0.61–4.28	0.14
No recurrence	Ref	-	-
Recurrence	0.61	−2.56–3.77	0.71

CI: confidence interval; coefficients with 95% CIs are nonstandardized and stand for points on the SHAI-14. The intercept represents the SHAI-14 score of the reference group (“TSH > 0.5” and “Low risk”, “0–1 or more comorbidities”, and “no recurrence”). TNM stage containing T4, T3, N1, and M1 was defined as “high risk” thyroid cancer; all others were classified as “low risk” thyroid cancer.

## Data Availability

The data contain personally identifying information, such as personal identity numbers, and potentially identifying information and disease data, and they are, therefore subject to ethical and legal restrictions on public sharing. We cannot share the dataset, which is on the individual level, because it is not permitted, according to the laws that apply in Sweden. The permission we obtained for this study (from the Regional Ethics Board, Sweden) only allowed public sharing at the group level.
